# Respiratory physiotherapy and incidence of pulmonary complications in off-pump coronary artery bypass graft surgery: an observational follow-up study

**DOI:** 10.1186/1471-2466-9-36

**Published:** 2009-07-28

**Authors:** Isabel Yánez-Brage, Salvador Pita-Fernández, Alberto Juffé-Stein, Ursicino Martínez-González, Sonia Pértega-Díaz, Ángeles Mauleón-García

**Affiliations:** 1 Physiotherapy Department, University of A Coruña, A Coruña, Spain; 2 Clinical Epidemiology and Biostatistics Unit, A Coruña University Hospital, Hotel de Pacientes 7a Planta, As Xubias, 84, 15006 A Coruña, Spain; 3 Cardiac Surgery Department, A Coruña University Hospital, A Coruña, Spain; 4 Nursing Unit, Transfusion Centre, Navarra, Spain

## Abstract

**Background:**

Heart surgery is associated with an occurrence of pulmonary complications. The aim of this study was to determine whether pre-surgery respiratory physiotherapy reduces the incidence of post-surgery pulmonary complications.

**Methods:**

Observational study of 263 patients submitted to off-pump coronary artery bypass grafting (CABG) surgery at the A Coruña University Hospital (Spain). 159 (60.5%) patients received preoperative physiotherapy. The fact that patients received preoperative physiotherapy or not was related to whether they were admitted to the cardiac surgery unit or to an alternative unit due to a lack of beds.

A physiotherapist provided a daily session involving incentive spirometry, deep breathing exercises, coughing and early ambulation. A logistic regression analysis was carried out in order to identify variables associated with pulmonary complications.

**Results:**

Both groups of patients (those that received physiotherapy and those that did not) were similar in age, sex, body mass index, creatinine, ejection fraction, number of affected vessels, O_2 _basal saturation, prevalence of diabetes, dyslipidemia, exposure to tobacco, age at smoking initiation, number of cigarettes/day and number of years as a smoker. The most frequent postoperative complications were hypoventilation (90.7%), pleural effusion (47.5%) and atelectasis (24.7%).

In the univariate analysis, prophylactic physiotherapy was associated with a lower incidence of atelectasis (17% compared to 36%, p = 0.01).

After taking into account age, sex, ejection fraction and whether the patients received physiotherapy or not, we observed that receiving physiotherapy is the variable with an independent effect on predicting atelectasis.

**Conclusion:**

Preoperative respiratory physiotherapy is related to a lower incidence of atelectasis.

## Background

Cardiac and abdominal surgery are associated with an occurrence of pulmonary complications (PC), defined as any pulmonary abnormality that occurs during the postoperative period which produces identifiable disease or dysfunction that is clinically significant, and which adversely affects the clinical course [1]. The incidence of complications depends on the localization of the surgery, the existence of risk factors and the criteria used to define them [2].

No single definition exists for PC during the postoperative phases [3]. However, those most frequently referred to in the literature include pneumonia, radiographic changes such as atelectasis or infiltrates, postoperative fever, respiratory failure and the prolongation of mechanical ventilation [4], as well as pleural effusions, pneumothorax and pulmonary oedema. The high incidence of PC is partly due to the alteration of normal ventilatory function, which is inherent to surgery carried out in the thoracic region [5].

Postoperative atelectasis is common in patients following coronary artery bypass graft surgery [6]. The cause of atelectasis is complex and may involve the contribution of numerous factors such as general anaesthesia, diaphragmatic dysfunction, abdominal distension, chest wall alterations, pleural effusions and pain [7].

Coronary bypass surgery with and without extracorporeal circulation results in the dramatic impairment of respiratory system mechanics [8]. Off-pump CABG does not provide greater protection from postoperative pulmonary dysfunction in comparison to CABG surgery with CPB [9]. The randomized controlled studies published to date have, on the whole, been incapable of conclusively demonstrating the advantages of off-pump CABG [10].

Furthermore, internal mammary artery (IMA) dissection may reduce blood supply to the ipsilateral intercostal muscles and the phrenic nerve, thereby leading to respiratory muscle dysfunction and, ultimately, atelectasis [11].

The use of the IMA for coronary artery bypass graft (CABG) surgery increases the percentage of PC in comparison to saphenous vein conduit bypass [12-15]. The appearance of atelectasis would appear to be inevitable during the postoperative phase [16].

Respiratory physiotherapy is regularly used to prevent or reduce PC following heart surgery, although there is hardly any documented evidence indicating the efficiency of these techniques [17]. Neither has consensus been reached regarding the most suitable and effective therapy [18].

Postoperative breathing exercises, combined with physical therapy after coronary artery bypass graft (CABG) surgery, have been reported to be as effective as physical therapy, including early mobilization on its own, in reducing atelectasis [19], pneumonia [20], or other types of pulmonary complications [21,22]. In a review, Paskina et al[23] concluded that evidence is lacking regarding the benefits of any type of prophylactic respiratory physical therapy following cardiac surgery, and that it is more comprehensive than has been justified by the findings of clinical research.

While most of the literature discusses interventions that have been used after surgery, a number of studies also exist on the effect of preoperative physical therapy on reducing PC after cardiac surgery. In 2005, Westerhal et al. [24] found that preoperative physiotherapy on the chest, including deep-breathing exercises, significantly decreased atelectasis and improved spirometry values, compared to a regime without breathing instructions following CABG surgery.

Other authors have studied short-term individual preoperative interventions. In a small, non-randomized study, Rajendran et al. [25] showed that preoperative short-term pulmonary rehabilitation in patients with chronic obstructive pulmonary disease who underwent CABG surgery decreased the incidence of atelectasis, consolidation, and pneumothorax, confirmed by chest roentgenogram, as well as reducing health care expenditure resulting from short ventilation times and hospital stay. Castillo and Haas [26] concluded that the use of preoperative and postoperative chest physiotherapy greatly reduced the number of patients who developed atelectasis, but did not have any significant effect on the number of patients who developed respiratory complications as a result of infection.

Preoperative physiotherapy before both abdominal and open-heart surgery has been found to diminish postoperative radiological alterations, auscultation, blood gases, and to improve quality of life [27-29].

Recently, Hulzebos et al. [30], in a randomized clinical trial of high-risk patients undergoing CABG surgery, found that intensive inspiratory muscle training (7 times a week, for at least 2 weeks before the day of surgery) reduced the incidence of PC and the duration of postoperative hospitalization.

Although some of these studies have demonstrated the efficacy of preoperative physiotherapy to prevent PC following CABG surgery, studies are lacking on the effect of preoperative physiotherapy on off-pump CABG surgery. The aim of this study is to determine whether preoperative respiratory physiotherapy in patients who undergo off-pump CABG surgery is related to the incidence of pulmonary complications.

## Methods

### Subjects

An observational study of 263 patients subjected to off-pump CABG surgery. The patients underwent surgery at the A Coruña University Hospital (north-western Spain). The inclusion criteria were: consecutive patients aged 18 and over, who underwent heart surgery during the study period (2005–2006). The exclusion criteria were: emergency surgery, patients subjected to on-pump CABG surgery, severe endocarditis, patients who had suffered strokes, reintervention and psychological disorders. Written informed consent was obtained from all of the patients, and the study was approved by the institutional review board/independent ethics committee (IRB/IEC).

One hundred and fifty-nine patients (60.5%) received preoperative respiratory physiotherapy, compared to 104 (39.5%) who did not. Patients who were admitted to the cardiac surgery unit received preoperative physiotherapy, while those who were admitted to another unit due to a lack of beds in the cardiac unit did not.

All of the patients were given a general anaesthetic. The philosophy of the A Coruña University Hospital is to carry out CABG surgery without manipulating the ascending aorta and without the need for on-pump, systematically resorting to the use of double mammary artery grafting (Tector technique) regardless of the patient's age [31], using median sternotomy. Mean surgery time was 3.36 ± 0.85 hours. During the postoperative phase, all of the patients were put on mechanical ventilation; 89 patients (32.2%) were extubated at the end of surgery and 175 (67.8%) received mechanical ventilation for a mean duration of 11.17 hours (SD = 18.40), and a median of 7 hours. No statistical differences were observed in the time on mechanical ventilation between patients who received preoperative respiratory physiotherapy and those who did not (Median: 6.0 vs. 7.2 hours, p = 0.14). All of the patients had pericardial and mediastinal drains and one or two pleural drains for at least 48 hours after surgery.

### Procedure

The preoperative protocol described below was restricted to the patients who received preoperative physiotherapy.

On the day of admittance to hospital, as well as explaining the regulations regarding the way the cardiac surgery unit worked to the patients, the nursing and auxiliary staff also gave them a flow-based incentive spirometer (IS) (Respiflo™ FS – Tyco Healthcare Group LP. Mansfield, MA 02048), explaining its use and insisting on the importance of using it every hour they were awake. The flow-based incentive spirometer (IS) (Respiflo™ FS) is a device based on a feedback principle. Patients can observe their movements during inspiration and/or expiration, which encourages them to keep up their efforts and to carry out sustained maximal inspiration.

On the morning after admittance, the unit physiotherapist met the patients who would be receiving preoperative physiotherapy. After assessment, the physiotherapist showed them the breathing exercises (BE) which consisted of: 10 deep breathing exercises, diaphragmatic, 30 lung-expansion manoeuvres at various levels (lower, mid and upper), with tactile stimulation, three-stage maximum inspiration, and 10 global lung expansion manoeuvres using the upper limbs, secretion-removal manoeuvres and supported/assisted coughing. The patients were encouraged to carry out 2 sessions on their own during the course of the day. Also, an explanation was given on how to use the flow-based incentive spirometer (Respiflo™ FS). The patients had to carry out 30 sustained slow maximal inspiratory (SMI) manoeuvres up to total lung capacity, followed by passive expiration to functional residual capacity (FRC). The aim of this procedure was to prevent the closure of the airways and alveolar collapse (5 series of 6 SMIs resting for between 30 and 60 seconds between each series each hour during the day whilst they were awake). They were also shown how to turn over in bed and how to sit up. During the session, patients were repeatedly informed of the importance of their cooperation in ensuring a rapid recovery.

During the postoperative phase, the exercises began on the morning after surgery. The patients were encouraged to carry out their BE with the help of the physiotherapist. The patients that did not receive preoperative physiotherapy were shown how to do the BE after assessment, and from then on all of the patients completed a daily physiotherapy session, under the supervision of the unit physiotherapist, for the rest of their hospital stay. In most cases, the patients were lifted from their beds into a chair by the nursing staff on the day following surgery (provided that they showed haemodynamic stability). They also walked around their room or short distances along the corridor 48 hours after surgery. On the third day, they were allowed to walk freely along the corridor.

A supervisor trained all of the physiotherapists before the start of the study. The supervisor was in charge of assigning patients to physiotherapists and controlling the time spent with each patient. The group of physiotherapists discussed the patients on a daily basis.

The following variables were studied in each patient: age (years), height (meters), weight (kg), BMI (weight/height^2^) (kg/m^2^), sex, previous pathology, tobacco use, functional grade (New York Heart Association (NYHA) Functional Classification), ejection fraction (%), pre- and postoperative peak expiratory flow (PEF) (L/min), pre- and postoperative physiotherapy sessions, pulmonary complications, pain and the degree of comprehension and surgical characteristics.

0_2 _saturation assessment (SpO_2_) was carried out via a non-invasive method using a pulse-oximeter (Model 9500. Nonin Medical Inc. Plymouth, MN, USA). It was observed that the pulse-oximeter readings had a good correlation with the direct arterial saturation reading when saturation was at around 80% (r = 0.878–0.879) [32]. The mean SpO_2 _of our test population was 96.56% (SD = 1.68%) during the preoperative phase and 95.69% (SD = 2.18) during the postoperative phase, breathing in atmospheric air when at rest.

Following the protocol of the A Coruña University Hospital for this type of surgical patients, a thoracic x-ray (anterior-posterior and lateral) was taken during the preoperative phase, as well as immediately after surgery in the intensive care unit (ICU), on the ward, once the drains had been removed (48 hours after surgery), and on discharge from hospital. X-ray examinations were given at the same frequency to patients who received preoperative physiotherapy and those who did not.

The intensivist used the thoracic x-ray to determine the presence or absence of complications during the patients' stay in the intensive care unit (ICU). Once they had been transferred to the ward, this task was carried out by the doctor in charge of the ward, who did not know if the patients had received preoperative physiotherapy or not. Patients were classified into the following groups according to the diagnosis of atelectasis: detectable or undetectable atelectasis by x-ray. Evidence of atelectasis on the X-ray were considered by the radiologists as direct signs (displacement of interlobar fissures) and indirect signs (local increase in density, elevation of diaphragm, mediastinal displacement and compensatory overinflation, displacement of hila, approximation of ribs, absence of an air bronchogram, absence of visibility of the interlobar artery) [33]. Atelectasis was classified by the radiologists as platelike (also called linear), and segmental (unilateral or bilateral lobar atelectasis). In order to increase the statistical power of the analysis, we reported the incidence of atelectasis as 'yes' or 'no', regardless of the degree. Atelectasis reported at any time throughout the follow-up was counted as an "event".

### Justification of the Sample Size

The sample size of n = 263 patients made it possible to study the characteristics of the patients with a security of 95% and a precision of ± 6.1% [34]. A sample size of n = 103 patients in each group would be required in order to detect a difference of 17% vs. 34% in the incidence of atelectasis between those patients receiving preoperative respiratory physiotherapy and those who did not, with a security of 95% and a statistical power of 80% [35]. Patients were included consecutively until the minimum sample size was achieved in both study groups. As a result, we finally included 159 patients who received preoperative respiratory physiotherapy, and 104 who did not.

## Data Analysis

A descriptive analysis was made of the variables included in the study. Quantitative variables are expressed as mean and standard deviation (SD). Qualitative variables are expressed as an absolute value and percentage, with a 95% confidence interval. The comparison of means was carried out using Student's t-test or the Mann Whitney test as appropriate. The association of qualitative variables was carried out using Chi-square statistics. A logistical regression analysis was carried out in order to determine which variables are associated with the presence of complications (atelectasis), adjusted by various covariables. The clinical relevance was estimated by calculating the Absolute Risk Reduction (ARR), the Relative Risk Reduction (RRR) between those who received physiotherapy and those who did not, and the number needed to treat (NNT) in order to prevent an event (atelectasis) [36].

The significance level was established at 0.05, and all p-values were two-tailed. Statistical analysis was carried out using the statistical software SPSS (Statistical Package for Social Sciences 14.0, SPSS Inc., 444 North Michigan Avenue, Chicago, IL 60611, USA) and EPIDAT 3.1 (Dirección Xeral Saúde Pública, Xunta de Galicia, Spain. Organización Panamericana de la Salud 525 23rd Street, N.W. Washington, D.C. 20037-3674 USA)

## Results

Mean age was 66.4 (SD = 9.4) years, with actual ages ranging between 34 and 86 years. There was a predominance of male patients (81.1%). Sixty-seven percent had hypertension, followed in order of frequency by dyslipidemia (63.6%) and diabetes (32.2%). In accordance with the inclusion criteria, all the patients (100%) had ischemic cardiopathy. Six point eight percent (6.8%) of the patients were smokers and 44.7% were former smokers; in other words, overall exposure to tobacco, either current or previous, was over 50%. Most of the patients had an NYHA functional grade of between 2 (41.4%) and 3 (46.6%). Most of the patients had 3 (64.6%) or 4 (6.3%) affected vessels, whilst 5.7% had 1 affected vessel and 13.3% had 2.

The characteristics of the patients included in the study, independently of whether they received preoperative physiotherapy or not, are shown in Table 1. In the case of those that did receive preoperative physiotherapy, the number of mean sessions was 2.2 (SD = 2.9). No significant differences were observed between those patients that received physiotherapy and those that did not in the following variables: age, sex, BMI, creatinine, ejection fraction, number of affected vessels, O_2 _basal saturation, prevalence of diabetes, dyslipidemia, exposure to tobacco, age at smoking initiation or the number of cigarettes per day and number of years as a smoker.

**Table 1:** Comparison of patients depending on whether they received preoperative respiratory physiotherapy or not.

	**Preoperative physiotherapy**				
		
	**Yes**		**No**		
	
	**Mean**	**SD**	**Mean**	**SD**	**p**
**Age (years)**	65.9	9.6	67.1	9.1	0.35
**Body Mass Index (BMI) (kg/m^2^)**	27.6	3.3	27.9	3.7	0.43
**Creatinine (mg/dL)**	1.1	0.3	1.1	0.3	0.68
**Affected vessels**	2.9	0.7	2.9	0.7	0.77
**Ejection fraction (%)**	59.7	14.2	59.7	14.7	0.99
**Number of cigarettes smoked per day**	23.3	13.7	20.0	12.7	0.64
**Age at smoking initiation (years)**	19.17	5.0	19.0	3.0	0.57
**Years as a smoker**	33.5	11.8	28.7	13.4	0.13
**O2 saturation (%)**	96.5	1.69	97.0	1.41	0.79

	**n**	**%**	**n**	**%**	**p**

**Sex**					0.29
Male	132/159	83.0%	81/104	77.9%	
Female	27/159	17.0%	23/104	22.1%	
**Dyslipidemia**	106/159	66.7%	61/104	58.7%	0.19
**Current exposure to tobacco**	13/159	8.2%	5/104	4.8%	0.56
**Diabetes Mellitus**	51/158	32.3%	33/104	31.7%	0.93

SD: standard deviation

The most frequent postoperative complications were elevation of the diaphragm (90.7%), pleural effusion (47.5%) and atelectasis (24.7%). The mortality rate was 1.1% (Table 2).

**Table 2:** Incidence of post-operative complications after surgery

**Variables**	**n**	**%**	**95% CI**
**Diaphragm elevation**	234/258	90.7%	87.0% – 94.5%
**Pleural effusion**	123/258	47.5%	41.2% – 53.8%
**Atelectasis**	64/258	24.7%	19.3% – 30.2%
**Pulmonary oedema**	21/258	8.1%	4.6% – 11.7%
**Pneumothorax**	6/257	2.3%	0.3% – 4.3%
**Pneumonia**	5/257	1.9%	0.6% – 4.5%
**Renal insufficiency**	3/259	1.2%	0.2% – 3.3%
**Neurological**	2/261	0.8%	0.1% – 2.7%
**Death**	3/263	1.1%	0.3% – 3.3%

CI: confidence interval

When comparing the pulmonary complications suffered by those that received physiotherapy with those that did not, the univariate analysis reveals that preoperative physiotherapy is associated with a lower incidence of atelectasis (17.3% vs 36.3%). (Table 3). The number of preoperative physiotherapy sessions was associated with the presence or absence of atelectasis. Patients with atelectasis had a lower number of preoperative sessions lower than patients without atelectasis (2.0 ± 3.5 vs 2.4 ± 2.7; p = 0.014). The presence of atelectasis was studied as an event throughout the follow-up, and most occurred 48 hours after surgery.

**Table 3:** Presence of complications with or without preoperative physiotherapy

	**Preoperative physiotherapy**						
				
	**Yes**		**No**				
	
	**n**	**%**	**n**	**%**	**p**	**RR**	**95% CI (RR)**
**Atelectasis**	27/156	17.3%	37/102	36.3%	0.01	0.48	0.31–0.73
**Pleural effusion**	75/156	48.1%	48/102	47.1%	0.87	1.02	0.79–1.33
**Pneumothorax**	4/156	2.6%	2/102	2.0%	0.75	1.31	0.24–7.01
**Pneumonia**	4/155	2.6%	1/102	1.0%	0.36	2.63	0.3–23.22
**Pulmonary Oedema**	16/156	10.3%	5/102	4.9%	0.12	2.09	0.79–5.53
**Diaphragm elevation**	140/156	89.7%	94/102	92.2%	0.51	0.97	0.90–1.05
**Renal insufficiency**	2/157	1.3%	1/102	1.0%	0.82	1.30	0.12–14.14
**Neurological**	1/157	0.6%	1/104	1.0%	0.77	0.66	0.04–10.47
**Wound infection**	3/158	1.9%	1/104	1.0%	0.54	1.97	0.21–18.73
**Sternal instability**	5/158	3.2%	0/104	0.0%	0.16	-	-
**Sternal dehiscence**	6/157	3.8%	0/104	0.0%	0.08	-	-
**Scar dehiscence**	3/158	1.9%	0/104	0.0%	0.28	-	-
**Death**	2/159	1.3%	1/104	1.0%	0.82	1.31	0.12–14.24

RR: relative risk; CI: confidence interval

In the univariate analysis, atelectasis is associated with the patients' sex and NYHA functional grade. Atelectasis was present in 21.8% of the male patients and 37.5% of the female patients.

The ejection fraction was slightly higher in those patients who did not have atelectasis in comparison with those that did (60.9% *versus *56.3%) (p = 0.08) (Table 4).

**Table 4:** Presence of atelectasis according to different variables

	**Atelectasis**				
		
	**Yes**		**No**		
	
	**Mean**	**SD**	**Mean**	**SD**	**p**
**Age (years)**	66.0	9.8	66.7	9.2	0.62
**Creatinine Clearance (MDRD) (mL/min/1.73 m^2^)**	77.4	24.4	75.0	22.1	0.53
**Creatinine Clearance (Cockcroft-Gault) (mL/min)**	75.9	22.1	73.5	24.2	0.54
**Body Mass Index (BMI) (kg/m^2^)**	27.7	3.4	27.7	3.5	0.97
**Ejection fraction (%)**	56.3	16.4	60.9	13.5	0.08
**Prior peak expiratory flow (PEF) (L/min)**	454.8	121.1	447.7	126.8	0.80

	**n**	**%**	**n**	**%**	**p**

**Sex**					0.02
Male	46/210	21.9%	164/210	78.1%	
Female	18/48	37.5%	30/48	62.5%	
**NYHA**					0.01
1	2/4	50.0%	2/4	50.0%	
2	4/24	16.7%	20/24	83.3%	
3	2/26	7.7%	24/26	92.3%	
4	3/3	100.0%	0/3	0.0%	
**COPD**					0.34
Yes	5/14	33.3%	9/14	66.7%	
No	59/242	24.4%	183/183	75.6%	
**Current smoker**					0.90
Yes	4/17	23.5%	13/17	76.5%	
No	60/241	24.8%	182/241	75.2%	
**Postoperative pain**					0.55
Yes	52/215	24.2%	163/215	75.8%	
No	12/42	28.6%	30/42	71.4%	

SD: standard deviation; NYHA: New York Heart Association (NYHA) Functional Classification; COPD: Chronic Obstructive Pulmonary Disease

After adjusting for age, sex, ejection fraction and whether physiotherapy was received or not, we can see that receiving preoperative physiotherapy was the variable which had an independent effect on the prediction of atelectasis (Table 5).

**Table 5:** Logistic regression model to predict atelectasis adjusted by various covariables

**Variable**	**B**	**p-value**	**OR**	**95% CI (OR)**
**Sex (Female)**	0.85	0.084	2.35	0.89 – 6.17
**Preoperative physiotherapy**	-1.15	0.004	0.32	0.14 – 0.69
**Age (years)**	- 0.02	0.294	0.98	0.94 – 1.02
**Ejection fraction (%)**	- 0.02	0.082	0.98	0.95 – 1.00
**Constant**	2.35	0.156		

B: regression coefficient; OR: odds ratio; CI: confidence interval

Clinical relevance in the prediction of the impact of preoperative physiotherapy reveals that the Relative Risk Reduction (RRR) was 52%, Absolute Risk Reduction (ARR) was 19% and the Number Needed to Treat (NNT) to prevent an event was 5 (Table 6).

**Table 6:** Clinical relevance in measuring the impact of preoperative physiotherapy and the presence of atelectasis

			**Preoperative physiotherapy**	
			
			**Yes**	**No**
**Postoperative Atelectasis**			17.3%	36.3%

		**95% CI**		

**RR**	0.48	0.31 – 0.73		
**RRR**	52%	24% – 70%		
**ARR**	0.19	0.08 – 0.29		
**NNT**	5	3 – 12		

CI: confidence interval; RR: Relative Risk; RRR: Relative Risk Reduction; ARR: Absolute Risk Reduction; NNT: Number Needed to Treat

## Discussion

This study shows that preoperative respiratory physiotherapy is significantly related to a lower incidence of atelectasis (17.3% vs 36.3%). Westerdahl et al. previously obtained this same result in CABG surgery in 2001, 2003 and 2005 [17,24,37].

In our study, the incidence of postoperative atelectasis in patients who received preoperative physiotherapy (17%) was similar to that detected by Johnson et al (1995) [38], who reported an incidence rate of 20%. Other studies have revealed lower incidence rates, such as those carried out by Stiller et al (1994) [21] with an incidence rate of 7.1%, or Jenkins et al (10%) [20]. In turn, Brasher et al (2003) discovered a complication rate amongst the preoperative physiotherapy group of 4.3%, compared to 2.6% in the case of the control group that did not receive preoperative physiotherapy [22]. The results obtained by Dull and Dull (1983) are much higher, who recorded 77% of patients diagnosed with pulmonary complications following open-heart surgery [39], and those of Westerdahl et al (2001) at 67% [17]. These differences may be attributable to numerous factors, although perhaps the most important are the varying criteria used to define the respiratory complications described in the studies.

Respiratory physiotherapy and deep breathing manoeuvres with or without mechanical devices are frequently used during the postoperative phase of heart surgery, and their impact on preventing PC is of little or no benefit [37]. Consequently, the deep breathing exercises designed to prevent PC following CABG surgery have been questioned [20,21]. However, body temperature, respiratory sounds and subjective symptoms have often been used in various studies to detect the presence of these complications, despite not being sensitive enough to detect slight or moderate pulmonary alterations. Even with thoracic x-rays, small pulmonary changes may go undetected. This may explain why previous studies have failed to prove the effect of respiratory exercises [37]. Westerdahl et al. (2003) [37] showed a significant reduction in the atelectasis area and an improvement in oxygenation following three series of 10 deep breathing exercises on the second day after surgery; changes that were detected using computerised axial tomography (CAT). As a result, it was possible to detect atelectasis that would not have been visible in conventional thoracic x-rays. Also, CAT scans make it possible to calculate atelectasis more precisely. We would also add that major atelectasis still remains after respiratory exercises. However, more efficient recruitment of the collapsed lung could improve the protective effects of physiotherapy [37].

Our results (which used conventional thoracic x-rays for diagnosis) also reveal that in the univariate analysis, pre-operative physiotherapy is substantially linked to a fewer cases of atelectasis (17% vs 36%). Additionally, as found in other studies [24], preoperative physical therapy did not modify postoperative SpO_2 _(95.8% vs. 95.4%). In the univariate analysis, atelectasis was associated with sex and the NYHA functional grade. Atelectasis was present in 21.8% of the men and 37.5% of the women. The ejection fraction was slightly higher in patients who did not suffer atelectasis than those who did (50.95% vs 56.29%), although this difference was not statistically significant (p = 0.08). In our study, the efficiency of preoperative physiotherapy was maintained after adjustments made according the variables shown above in a multivariate analysis. This efficiency is not only revealed by the significant finding (p < 0.05) that pre-operative physiotherapy prevents atelectasis, but also by the size of the difference and clinical relevance. This clinical relevance can be seen clearly from the Relative Risk Reduction of 52%. This means that preoperative physiotherapy reduced the risk of atelectasis by 52% when compared to those patients who did not receive it. Absolute Risk Reduction (ARR) was 19%, meaning that out of every 100 patients who received physiotherapy, 19 cases were prevented. The Number Needed to Treat (NNT) with preoperative physiotherapy was 5, which means that for every 5 people we treated with preoperative physiotherapy, 1 case of atelectasis was prevented.

Although Westerdahl et al (2003) obtained their results with 30 voluntary deep breaths; they state that they do not know whether efficiency would be improved by increasing the frequency and intensity of the exercises [37]. They acknowledge that a mechanical device could help patients to remember to carry out the respiratory exercises, and that patients find these devices both useful and motivating. As previously mentioned, in our case the patients used a flow-based incentive spirometer (Respiflo™ FS) and carried out 30 slow SMI manoeuvres every hour they were awake, as well as a daily deep breathing session supervised by the unit physiotherapist.

In this case it is important to stress that showing the patient how to use the IS during the preoperative phase is essential in order to guarantee its effective use during the postoperative stage [4].

Clearly, in an ideal situation a randomised clinical trial should be carried out in order to demonstrate the efficiency of an intervention. This study describes the efficiency of an intervention in a study carried out within the context of standard medical practice, in which the patients from both groups were comparable in several variables of interest including age, BMI, creatinine, ejection fraction, the number of affected vessels and O_2 _basal saturation. Neither were there any differences in sex, prevalence of diabetes, dyslipidemia and exposure to tobacco, age at smoking initiation, nor the number of cigarettes smoked per day and number of years as a smoker.

Other limitations could be a lack of assessment of patient compliance, which could result in a bias toward the null hypothesis of 'no difference', and decrease the effect of the physiotherapy. This possible bias implies that the effect of physiotherapy could be even greater.

CAT scans are not routinely performed after surgery, meaning that as this study was carried within the context of standard medical practice, the patients did not receive CAT scans. If there was an underestimation of the incidence of atelectasis, this underestimation should be present in both groups of patients. This underestimation could decrease the incidence of atelectasis, although it would not affect the differences detected between the two groups.

Although the efficiency of physiotherapy in preventing atelectasis has been described in a number of publications [17,24-26,37], the relevance of routine physiotherapy is still questioned in patients who have not undergone complicated cardiac surgery [20,21]. Meta-analysis presented in the literature suggests that in patients undergoing heart surgery without complications, the use of physiotherapy during the post-intubation period does not provide additional benefits to their postoperative evolution [40]. This study evaluates the effects of respiratory physiotherapy in patients receiving off-pump coronary bypass surgery, using the mammary artery as a graft.

As already mentioned, in deciding to carry out this study into the efficiency of preoperative physiotherapy in the prevention of pulmonary complications, we simply took advantage of existing circumstances, in the light of the comparability of the groups of patients who received preoperative physiotherapy and those who did not. The results from this study should be confirmed by randomized clinical trials.

## Conclusion

This study shows that preoperative physiotherapy (involving incentive spirometry, deep breathing exercises, assisted coughing and early ambulation) after off-pump CABG surgery is related to a lower incidence of atelectasis (17% vs 36%), a difference which may be considered as being both significant and clinically relevant.

## Competing interests

The authors declare that they have no competing interests.

## Authors' contributions

SPF and SPD participated in the design of the study, performed the statistical analysis and contributed to draft the manuscript. IYG, UMG and AMG participated in the design of the study and the gathering of data. AJS contributed to the design of the study, collaborated in gathering of data and helped to draft the final manuscript. All authors read and approved the final manuscript.

## Pre-publication history

The pre-publication history for this paper can be accessed here:


